# Combined serratus anterior and latissimus dorsi myocutaneous flap for obliteration of an irradiated pelvic exenteration defect and simultaneous site for colostomy revision

**DOI:** 10.1186/1477-7819-12-319

**Published:** 2014-10-22

**Authors:** Masaki Fujioka, Kenji Hayashida, Sin Morooka, Hiroto Saijo, Takashi Nonaka

**Affiliations:** Department of Plastic and Reconstructive Surgery, National Hospital Organization Nagasaki Medical Center, 1001-1 Kubara 2, Ohmura City, zip 856-8562 Japan; Department of Surgery, National Hospital Organization Nagasaki Medical Center, 1001-1 Kubara 2, Ohmura City, zip 856-8562 Japan

## Abstract

**Background:**

Usually, several surgical methods are used, with re-suturing, free skin grafting and local flaps, for the reconstruction of wall defects after abdominoperineal resection. However, or larger defects, free flaps have been preferred because they can provide a large area of well-vascularized soft tissue, which is suitable for defect repair. We present the case of a large abdominal wall defect, which was treated with a free combined serratus anterior and latissimus dorsi myocutaneous flap, resulting in a successful outcome.

**Case presentation:**

A 38-year-old female originally had squamous cell carcinoma of the cervix uteri, and had undergone radical hysterectomy and oophorectomy followed by radiotherapy. She had a recurrence of the cervical cancer after 13 years, and underwent pelvic exenteration. However, the mid-abdominal wound developed dehiscence and an abdominal full-thickness defect communicating with the pelvic cavity. Furthermore, the adhered colon developed necrosis, which drained stools into the pelvic cavity, resulting in chronic peritonitis. During surgery, the empty pelvic cavity was filled with a combined serratus anterior and latissimus dorsi myocutaneous flap to prevent chronic peritonitis, to create a new stoma in the skin paddle of the flap for the necrotic colon, and to separate the pelvic cavity from the drained stools. The patient could walk in the absence of abdominal hernia formation and relapse of infection.

**Conclusions:**

A combined serratus anterior and latissimus dorsi myocutaneous free flap was applied to cover the raw surface and reinforce the abdominal wall and to fashion a new colostomy, as well as successfully filling the pelvic cavity with a large muscle body and long vascular pedicle. This is the optimal method for reconstructing severe abdominal wall defects that have many complications.

## Background

Reconstruction of a wall defect after abdominoperineal resection is very demanding with regard to its functional outcome. Several surgical methods are used, with re-suturing, free skin grafting and local flaps, which can be useful when the defect is relatively small [1]. However, for larger defects with further complications, such as the formation of a colostomy or iliac conduit, and wound infection, a large free flapis usually required to repair the abdominal wound [2–4].

We present the case of a colon fistulae and pelvic exenteration abdominal wall defect, treated with a free combined serratus anterior and latissimus dorsi myocutaneous flap, along with a new colostomy fashioned in a free flap, resulting in a successful outcome.

## Case presentation

**Figure 1:**
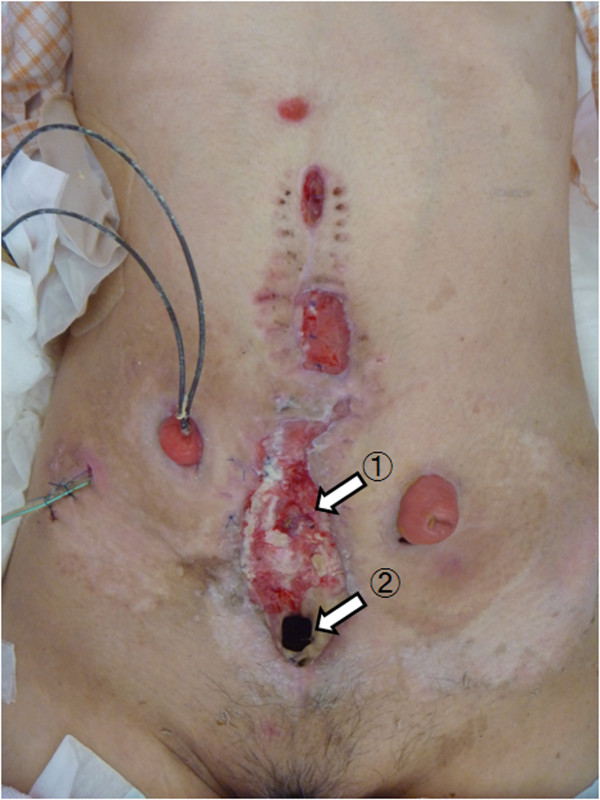
**Abdominal wound.** The ruptured colon at the center of the abdominal cavity (1), and the fistula penetrating the pelvic cavity (2) are shown.

**Figure 2:**
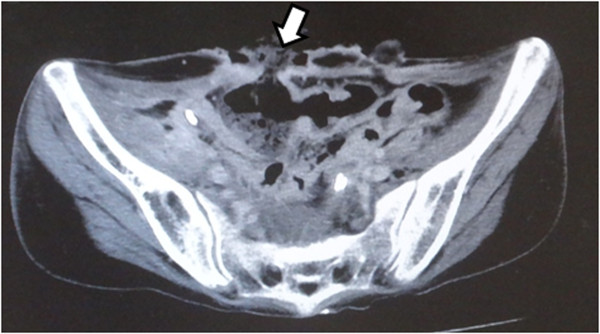
**Computed tomography scan showing the necrotic colon (arrow).**

**Figure 3:**
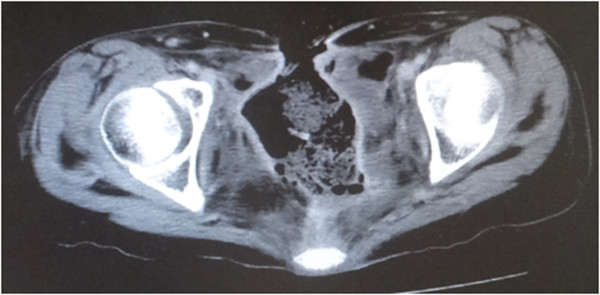
**Computed tomography scan.** The pelvic cavity, which extended from the pubic symphysis to the coccyx internally and communicated, is shown.

**Figure 4:**
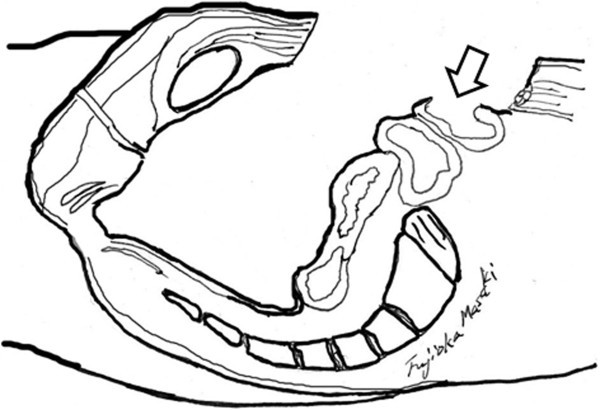
**Sagittal section showing the necrotic colon (arrow) and fistula pelvic cavity.**

A 38-year-old female originally had squamous cell carcinoma of the cervix uteri, and had undergone radical hysterectomy and oophorectomy followed by post-operative chemotherapy and radiotherapy. After a disease-free period of 13 years, the cervical cancer recurred, and she underwent pelvic exenteration including the bladder, rectum, sigmoid colon and vagina. An end colostomy and ileal conduit were fashioned. However, her post-operative course was complicated by small bowel necrosis, which required another laparotomy to remove it. The mid-abdominal wound developed dehiscence. The pelvic cavity, which extended from the pubic symphysis to the coccyx internally and communicated with the perineal defect measuring 8 × 6 cm, was packed with saline-soaked gauze dressing every day. The remaining bowel and omentum were adherent at the center of the abdominal cavity, perhaps due to the previous radiation. Furthermore, the adhered colon developed necrosis, which drained stools into the pelvic cavity, resulting in chronic peritonitis (Figures 1, 2, 3 and 4).

**Figure 5:**
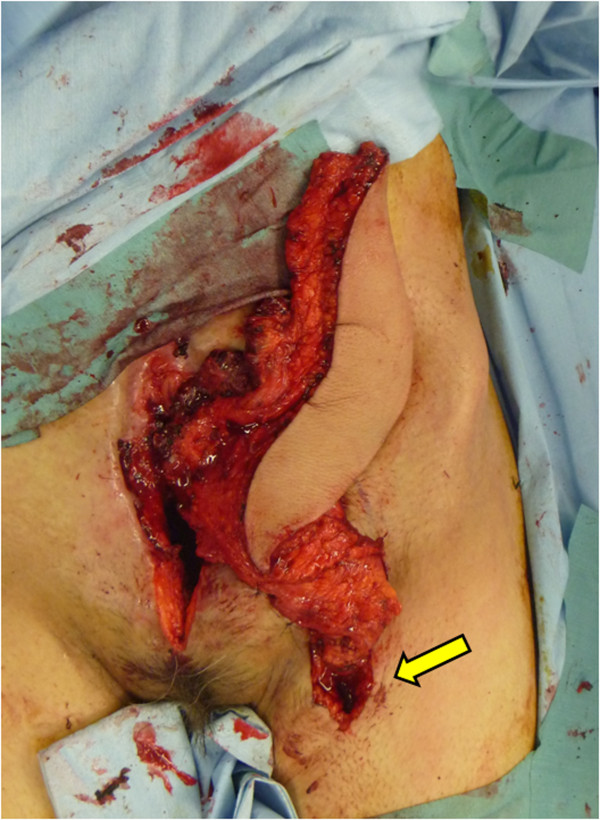
**Intra-operative view.** Muscle flaps have been inserted into the pelvic cavity, and the thoracodorsal vessels have been connected with a branch of the deep vessels of the thigh (arrow).

**Figure 6:**
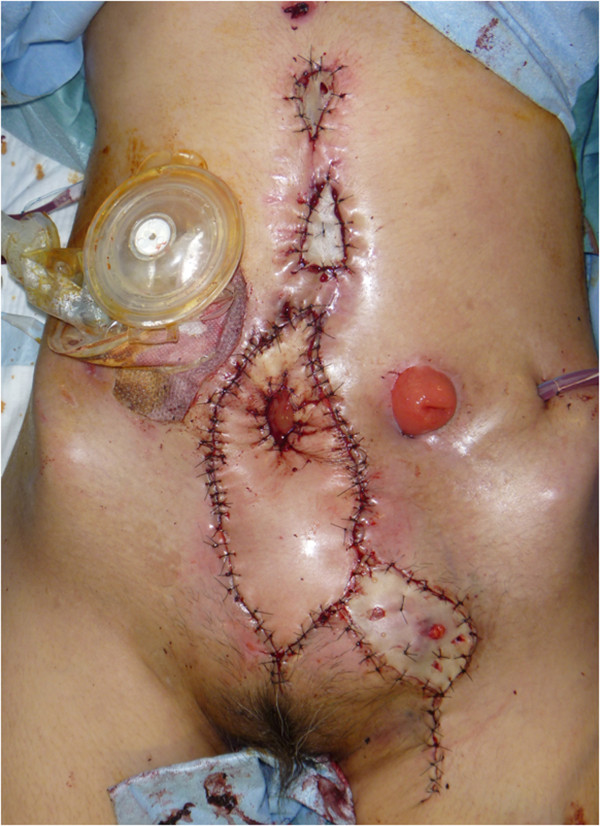
**Intra-operative view.** A skin paddle has been applied to cover the abdominal fistula, and a new colon stoma fashioned through the slit made in the skin flap.

**Figure 7:**
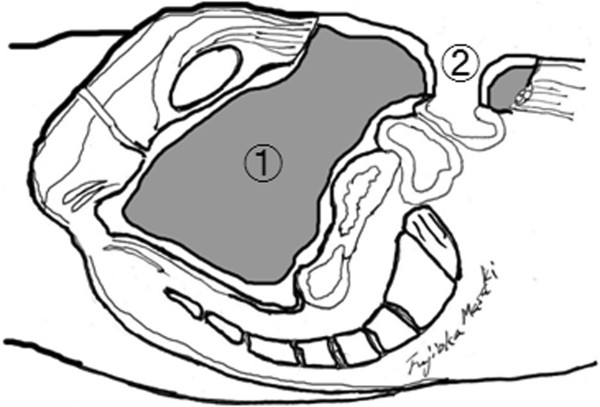
**Sagittal section.** The pelvic cavity is filled with muscle (1) and a new colon stoma has been fashioned in the skin flap (2).

Surgery was planned so that the empty pelvic cavity could be filled with a large vascularized muscle to prevent chronic peritonitis, to create a new stoma for the ruptured colon and to separate the pelvic cavity from the drained stools. At first, the abdominal full-thickness defect combined with its communication with the pelvic cavity was de-epithelialized and curetted carefully. The patient was then placed in a right lateral decubitus position, and a left combined serratus anterior and latissimus dorsi myocutaneous flap with a 25 × 7-cm elliptical skin island, both of which were based on the thoracodorsal vessels, was harvested in the standard manner.Following the primary closure of the donor defect, these muscle flaps were inserted into the pelvic cavity. Then, the thoracodorsal artery was connected by end-to-end anastomosis with a branch of the profunda femoris artery, and two thoracodorsal veins were connected by end-to-end anastomosis with the branches of the vena comitante of this profunda femoris (Figure 5). Finally, a skin paddle was applied to cover the abdominal fistula, and a new colon stoma was fashioned through the slit made in the skin flap (Figures 6 and 7).

**Figure 8:**
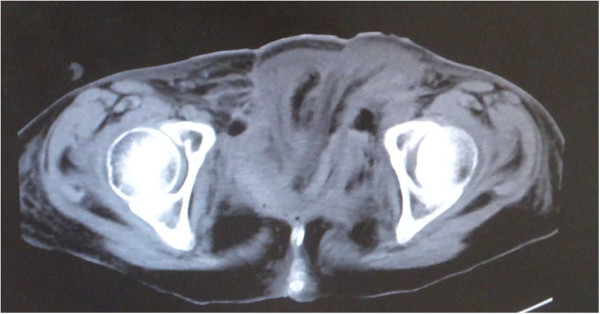
**Computed tomography scan after 2 weeks showing the pelvic cavity filled with the transported muscle.**

**Figure 9:**
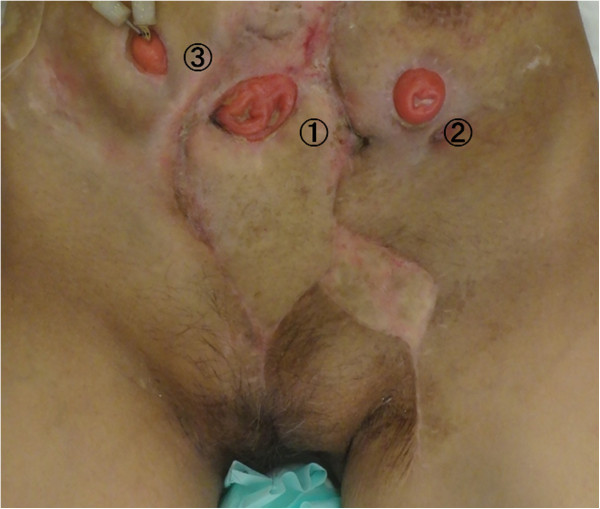
**Abdominal wall 3 months after surgery.** There is favorable coverage of the wound and a new colon stoma fashioned on the flap (1). Also shown are conventional stoma (2) and conventional urinary-stoma (3).

A computed tomography scan taken after2 weeks showed that the pelvic cavity had been filled with the transported muscles (Figure 8). She underwent excess free skin grafting due to partial necrosis developing at the distal end of the skin flap 3 weeks later. Three months later, the patient could walk in the absence of abdominal hernia formation and relapse of infection (Figure 9).

### Ethical considerations

The authors herewith certify that they are responsible for the contents of the manuscript. They have complied with the guidelines for conducting research with human subjects. The procedures are in accordance with the ethical standards of our institutional committee on human experimentation.

## Conclusions

Simple wall defects after abdominoperineal resection due to skin marginal necrosis and seroma development have sometimes been reported, and these small defects can be closed with simple methods, including debridement and direct re-suture, the component separation method, and with pedicled muscle or fasciocutaneous flaps [1, 5].

A pedicled tensor fascia lata fasciocutaneous flap has usually been used for abdominal defects [6]. However, large full-thickness abdominal wall losses caused by gunshot wounds, severe infection such as necrotizing fasciitis and intra-abdominal sepsis, or en bloc excision of neoplasms, require free flap transfer for reconstruction, because the distal third of the tensor fascia lataflap is at risk of necrosis unless a delaying procedure is included [2–4]. A pedicled anterolateral thighflap may serve as a good portion for the reconstruction of a lower abdominal wall defect, and it is also available for whole abdominal wall defect restoration if it is applied as a free flap. The advantages of this flap are that it can be harvested as a musculocutaneous flap with the vastus lateralis muscle to fill a tissue defect, and the lack of a need for changing position facilitates harvesting of the flap [7, 8]. However, the vastus lateralis muscle is too small to fill the large pelvic cavity, and harvesting a large amount of the vastus lateralis carries a high risk of morbidity at the donor site [9].

Reconstruction of the major defect in the abdominal wall in our case led to several problems, including: a non-healing wound with infection due to irradiation, a large dead space due to pelvic exenteration, and continuous contamination of the pelvic cavity due to stools from the ruptured bowel. First, a free flap transferwas required to treat these large irradiated wounds, because the tissue surrounding the ulcer crater has often been compromised by radiotherapy. Thus, if the reconstruction were performed with a local flap, it would have resulted in the loss of at least part of the flap [10]. Furthermore, a free flap is supplied by large blood vessels, which may promote the healing process in irradiated tissue [11]. When performing a free flap transfer around an irradiated area, identifying an acceptable recipient vessel is not always easy. Chronic endothelio-angiitis in recipient vessels caused by radiation may be one of the factors leading to thrombosis [12]. So, it is important to select a flap with a long pedicle, as the suitable recipient vessel may be distant from the wound. In our case, the inferior epigastric vessels, the first choice for a recipient vessel, collapsed and did not supply sufficient blood flow due to the radiation damage. Thus, there was further vessel dissection until our realization that the profunda femoris artery provided a sufficient amount of blood.

Second, restoration of abdominal muscle defects requires synthetic materials or a flap to prevent a hernia. The insertion of synthetic materials has been reserved for large defects of the abdominal wall, but they have proven increased complication rates, especially in contaminated wounds [2, 6]. Therefore, a myocutaneous free flap is desirable. The close continuity of the remaining rectus abdominis muscle after debridement and the muscle in the transferred flap will prevent the formation of hernias [3]. Third, our case required a durable skin component to make a new stoma, and a large area of soft tissue to occupy the large pelvic dead space.

To resolve these problems simultaneously, a combined serratus anterior and latissimus dorsi myocutaneous free flap was applied to cover the raw surface, to reinforce the abdominal wall and to fashion a new colostomy, as well as filling the pelvic cavity with a large muscle body with a long vascular pedicle. This is the optimal method for reconstructing severe abdominal wall defects associated with many complications.

## Consent

Written informed consent was obtained from the patient for publication of this case report and any accompanying images. A copy of the written consent is available for review by the Editor-in-Chief of this journal.
